# Validity and reliability of Veloflex to measure active cervical range of motion in asymptomatic and symptomatic subjects

**DOI:** 10.7717/peerj.11228

**Published:** 2021-04-05

**Authors:** Germán Cánovas-Ambit, José A. García-Vidal, Rodrigo Martín-San Agustín, Aurelio Arenas Dalla-Vecchia, Mariana Sánchez-Barbadora, Francesc Medina-Mirapeix

**Affiliations:** 1 Department of Physiotherapy, University of Murcia, Murcia, Spain; 2 Department of Physiotherapy, University of Valencia, Valencia, Spain; 3 Department of Electromagnetism and Electronics, University of Murcia, Murcia, Spain

**Keywords:** Cervical, Range of motion, Validity/reliability, Optoelectronic device

## Abstract

**Background:**

Neck pain has a high annual incidence and decreases the cervical active range of motion (ROM). Clinicians use various methods to evaluate cervical range of motion (CROM) that some of them have also been proposed to give instant feedback. Accordingly, this study aimed to examine the validity and reliability of Veloflex (VF) to measure the CROM by comparison with the cervical range of motion (CROM) device, and to examine their test-retest reliability.

**Methods:**

Thirty-eight healthy and 20 symptomatic participants were evaluated. Cervical flexion-extension, side bending, and rotations were tested in two sessions, first by the CROM and VF and in the second only with the VF. To evaluate the concurrent validity and agreement between CROM and VF, Pearson correlation coefficient (*r*) and Bland–Altmann plots were used. Reliability were evaluated using intra-class correlation (ICC), standard error of measurement (SEM) and minimal detectable change (MDC).

**Results:**

CROM and VF showed excellent correlation for all movements (*r* > 0.960). Both devices provided small mean ‘bias’ (≤1.29%) in all movements regarding CROM measures. The intra-rater and inter-rater reliability of the VF was excellent (ICC > 0.98). SEMs ranging from 0.72% to 2.38% and the MDC ranging from 1.22° to 2.60° in all participants. The results support the validity and reliability of VF to measure CROM. For its use, with a basic training is enough to get reliable measurements.

## Introduction

Neck pain has a high annual incidence (range between 10.4% and 21.3%) in the general population, with a higher incidence in office and computer workers (Hoy et al., 2010; Blanpied et al., 2017). The presence of neck pain usually decreases the cervical active range of motion (ROM) (Hagen et al., 1997; Stenneberg et al., 2017). Thus, cervical range of motion (CROM) assessment plays an important role in clinical examination of the neck region (Williams et al., 2010; Reese, Bandy & Yates, 2017) and its restoration is one of the objectives recommended by the clinical practice guidelines from the Orthopaedic Section of the American Physical Therapy Association (Childs et al., 2008).

There are a wide variety of methods to evaluate CROM, ranging from more traditional methods (simple visual estimation, inclinometers, or goniometers) (Youdas, Carey & Garrett, 1991; Audette et al., 2010) to more recent devices such as smartphone applications (Tousignant-Laflamme et al., 2013; Quek et al., 2014; Pourahmadi et al., 2018; Stenneberg et al., 2018; Rodríguez-Sanz et al., 2018) or optoelectronic devices (Zhongyang et al., 2017; Feng et al., 2019). A recent systematic review of reliability and validity studies of CROM measurement methods concluded that the CROM device (composed of three inclinometers) was shown to be clinimetrically sound (Williams et al., 2010). Others like the Spin-T goniometer and the single inclinometer also have a “good” reliability and validity (Williams et al., 2010). Instead, universal goniometer would not be recommended to measure CROM due to the difficulty in locating anatomical landmarks to use as a reference point and the evaluation of dynamic cervical movements (Keogh et al., 2019). More recent studies also show stability for the use of different mobile applications to measure CROM (Tousignant-Laflamme et al., 2013; Quek et al., 2014; Rodríguez-Sanz et al., 2018). Even so, the reliability of mobile applications is influenced by the smartphone model used, limiting its use to specific models. In addition, these methods have commonly been examined for asymptomatic patients, so the application of these results to patients should be done cautiously (Blanpied et al., 2017).

Some of these methods that instantaneously quantify position have also been proposed to give ROM feedback to patients, which has been shown to be beneficial for CROM improvement (Park et al., 2017; Ribeiro & Silva, 2019). While mobile applications have been used to give feedback especially of neck movements (Park et al., 2017), optoelectronics devices (i.e. ROM monitoring by marker tracking) have been used in other regions (Imam et al., 2013).

Veloflex (VF) is a new optoelectronic device developed to give instantaneous measures of movement, providing ROM and visual feedback through a computer application. This system has the advantage of being highly sensitive to movement when using a high definition camera, improving comfort to the subject by only needing markers that are attached to the skin. Thus, this study aimed to validate the VF to measure CROM (flexion-extension, side bending and rotations) comparing with the CROM device as the gold standard, and to examine their test-retest reliability.

## Materials and Methods

### Subjects

Thirty-eight subjects without cervical mobility disorder (asymptomatic subjects) and twenty subjects with cervical mobility disorder (symptomatic subjects) were recruited. Asymptomatic subjects were selected from volunteers who had no history of head or neck pain (Visual Analog Scale (VAS) = 0) and have more than 75% of normal ROM (Swartz, Floyd & Cendoma, 2005) for all cervical movements. Symptomatic subjects were included if they reported limitation of any cervical movement, either due to an average pain of ≥ 3/10 at rest measured by the VAS or reported vertigo of ≥3/10 measured by the Visual Vertigo Analogue Scale (VVAS) lasting longer than 1 week (Rutledge et al., 2013; Dannenbaum, Chilingarian & Fung, 2019; Carrasco-Uribarren et al., 2021). They were excluded if they presented possible red flags, vascular problems, or a clinical history of previous vestibular disorders different to a benign paroxysmal positional vertigo (for patients with vertigo) (Rushton et al., 2014). All those who initially contacted the researchers to participate in the study were ultimately examined. All subjects provided written informed consent. The institutional review board of the University of Murcia approved the study (CEI-2263), following the ethical criteria established in the protocol.

### Measurement instruments

**Figure 1:**
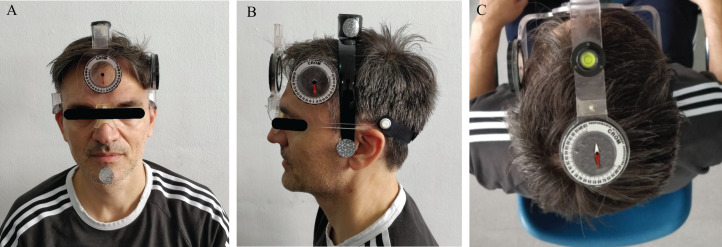
CROM’s placement for measurement for frontal (A), sagittal (B) and transverse (C) plane movement analysis. Frontal, lateral and superior inclinometers allow to evaluate the CROM during side bending, flexion/extension, or rotation, respectively. Photo credit: Rodrigo Martín-San Agustín

Cervical range of motion device (Performance Attainment Associates, Roseville, MN, USA) consists of a plastic frame mounted on the subject’s head similarly to wearing optical glasses. This measurement system involves several anchors and three inclinometers located in three different areas to measure the ROM in sagittal, frontal and transverse planes. The first anchor consists of an arm placed on the bridge of the nose and ears like glasses, closed with an arch that fixes it to the occipital area. A second arm is placed in C7, which was located by asking subjects to flex, and extend followed by cervical rotation while palpating for the most mobile segment on the cervicothoracic junction region (Palmer & Epler, 1990). The embedded bubble inclinometer on this arm was used to ensure verticality. A magnetic collar is placed over the shoulder to take into account any rotation of the trunk. The two inclinometer dials that measure flexion/extension (sagittal plane) and side bending (frontal plane) are dependent on gravity. The third inclinometer was on the top part of the head measures the cervical rotation (transverse plane), measuring by means of its magnetic dependence to the collar (Fig. 1). This instrument considered the gold standard (Tousignant-Laflamme et al., 2013; Kubas et al., 2017; Rodríguez-Sanz et al., 2018; Feng et al., 2019), as its validity and reliability have been extensively studied (Tousignant et al., 2000, 2006; Lee, Nicholson & Adams, 2004; Audette et al., 2010; Carvalho et al., 2014).

**Figure 2:**
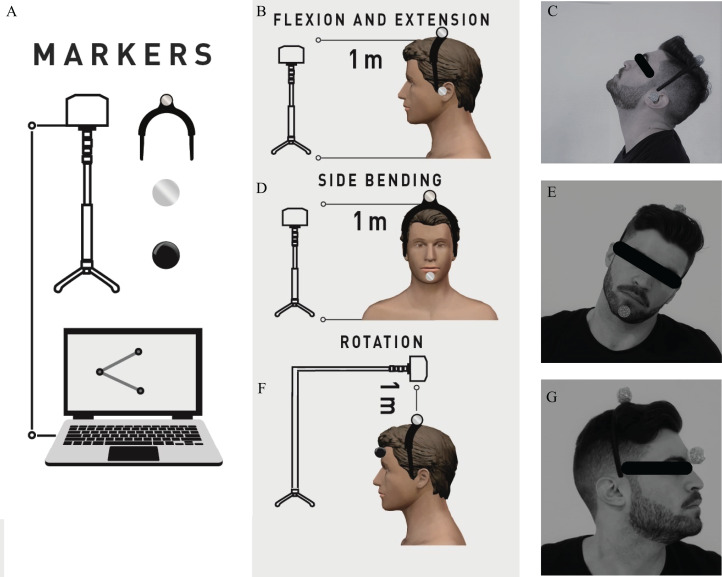
Veloflex setup (A) and participant and Veloflex positions for sagittal (B and C), frontal (D and E) and transverse (F and G) plane movement analysis. Veloflex was located at the height (frontal and sagittal planes) or above (transverse plane) of the head, and by tracking the markers, a laptop software computed and recorded the different ROM values. Photo credit: Rodrigo Martín-San Agustín

Veloflex (Deportec, Murcia, Spain) is an optoelectronic system designed to measure ROM and feedback from it. As previously described in Medina-Mirapeix et al. (2019), VF includes three elements: an infrared camera on a tripod, a laptop, and markers to place on the skin. The camera is placed on the adjustable tripod at a distance of 1–1.5 m from the participant and is located at the height of the joint to be examined. By tracking the markers, VF can determine the angle formed by two bone segments (taking three reference points: a joint axis and two references), or between a bone segment and the horizontal/vertical line drawn on the joint axis. While for flexion/extension and side bending, the camera captures ROM from a frontal view, for rotation this record is from a superior view (Fig. 2). Two VF markers were used for each movement, one common for all movements located by means of a rigid headband in the center of the head. The second marker was placed on the ear for movements in the sagittal plane, on the chin for movements in the frontal plane, and in the center of the forehead for movements in the transverse plane.

The constant capture of the movement can be used to quantify the ROM or give feedback instantly from it. Depending on the proximity of the camera to the joint, the accuracy of the system has been reported between 0.1° and 1° (Peña García-Orea et al., 2018). It has been previously validated to assess ROM in joints of the upper and lower limb, showing excellent intra- and inter-examiner reliability (Medina-Mirapeix et al., 2019; Martín-San Agustín et al., 2019).

### Procedures

**Figure 3:**
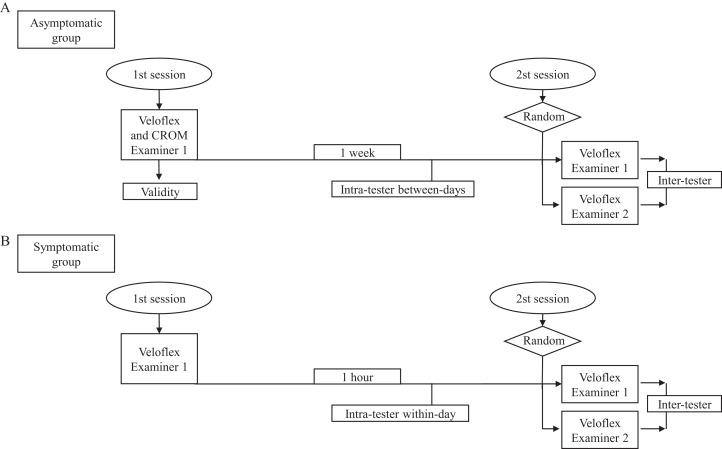
Flow chart represents a process of the study for both asymptomatic group (A) and symptomatic group (B).

For concurrent validity, all CROMs were simultaneously measured by the CROM and VF, except rotations (measured 5 min apart because they were not possible together). Intra-rater reliability was explored for both groups, between-days reliability for the asymptomatic group and within-day reliability for the symptomatic group (Fig. 3). Thus, an examiner (physiotherapist experienced in the VF and CROM) evaluated the participants in two sessions, with a 1-week interval between sessions for asymptomatic participants and with 1-h interval between measurements (to minimize the influence of their changes in symptoms on the device’s reliability) for symptomatic participants. For inter-rater reliability, a second examiner (newly graduated physiotherapist) without experience was instructed in the procedures and performed the same tests in the second session (Fig. 3). A research assistant recorded ROM values in order to avoid bias, reading the C-ROM and recording the evaluation of the VF’s software. Both examiners were unaware of the other’s scores. The order of examiners and tests were randomized.

The testing protocol was strictly identical for the two sessions. All tests required subjects sit on a chair. The VF was placed by physiotherapist (an experienced or inexperienced examiner) on adjustable tripod stand to 1 m distance away from participant and located at the height or above (for rotations) of the head (Fig. 2). Test positions and procedures were chosen based on goniometry manuals (Norkin & White, 2016). To test the movements, the physiotherapist placed the CROM and the markers on the skin and/or a head arch, and trained subjects using standardized instructions for each movement: directions, stabilizations required (examiner’s hands on subjects’ shoulders), pauses, repetitions and sequence. For symptomatic subjects it was asked that the movements did not have to cause pain or dizziness. Afterwards, subjects carried out five full movements to familiarize and warm-up tissues. After warm-up, VF was on and subjects were asked to move from the initial neutral position toward maximal range in a given primary direction, hold it three seconds and return to neutral position. The specific instructions for each movement were: (1) for flexion, “I would like you to tuck your chin then move your chin to your chest without poking your chin forward”, (2) for extension, “I would like you to look up toward the ceiling without letting your back lose contact with the chair”, (3) for side bending, “I would like you to keep your chin tucked and bring your left ear to your left shoulder, and then your right ear to your right shoulder. Please keep your shoulders down”, and (4) for rotation, “I would like you to keep your chin tucked, your eyes level and look over your left shoulder, then your right shoulder” (Kubas et al., 2017). After a 5 s pause this movement pattern was repeated twice for a total of three trials per movement pattern. After five seconds, the opposite direction was measured similarly. There was a 60-s rest period between cardinal planes in order to change camera position and markers.

### Statistical analysis

Participant’s characteristics and ROMs were summarized using means and SDs or percentages, as appropriate. Validity is defined as the extent to which the method measures what it is intended to measure (Streiner & Norman, 2008). To evaluate the concurrent validity between the measurements of the two devices (VF and CROM), the Pearson’s product-moment correlation coefficient (*r*) was used (Lin & Yao, 2014). Validity is defined as the extent to which the method measures what it is intended to measure (Martín-San Agustín, Sánchez-Barbadora & García-Vidal, 2020). To evaluate the agreement between devices, Bland–Altman plots were created and were calculated: upper and lower limits of agreement (LoA), the mean and the standard deviation of the difference between the two devices (these concepts were called ‘bias’ and ‘imprecision,’ respectively) (Giavarina, 2015), and their respective percentage with respect to the CROM values. A priori, we established the acceptable LoA needed to recommend the new measurement system to be substituted for the previously used device. These limits were ±2.5° concerning CROM because this magnitude is the minimal standard error of measurement (SEM) of this device across the six movements (Fletcher & Bandy, 2008).

Relative reliability is the degree to which individuals maintain their position in a sample with repeated measurements, which was assessed using intraclass correlation coefficients (ICCs) (Streiner & Norman, 2008). Absolute reliability is the degree to which repeated measurements vary for individuals, expressed as the SEM and calculated as $SDx\sqrt{1-ICC}$, where SD is the SD of all measures from the subjects. We also calculated the minimal detectable change (MDC) with a 95% confidence interval, calculated as SEMx1.96x√2. The reliability was classified as excellent (ICC > 0.90), good (ICC = 0.76–0.90), moderate (ICC = 0.51–0.75) and poor (ICC < 0.50) (Martín-San Agustín et al., 2020). All statistical analyses were performed using SPSS version 24.0.

Sample sizes for the asymptomatic and symptomatic subjects were calculated to determine whether the expected ICCs were significantly higher than 0.5 (with two sessions per participant) assuming a significance level of 5% and power of 90%. For asymptomatic subjects, the expected ICC was set at 0.8, since outcome variables were unknown, and the sample size was 30 participants. For symptomatic subjects, the expected ICC was increased to 0.9 since ICCs from asymptomatic were already known, the required number of subjects was 12 subjects. The WINPEPI program was used for these estimates (Abramson, 2011). However, ultimately, we included 5–10 more symptomatic subjects in the final sample in order to increase the study power.

## Results

### Subjects

Asymptomatic subjects had an average age of 34.1 years with a BMI of 24.4 kg/m^2^ and symptomatic subjects had an average age of 42.8 years with a BMI of 24.7 kg/m^2^ (Table 1). Thirteen subjects presented limitation of CROM associated with pain (VAS = 4.6) and seven subjects associated with vertigo (VVAS = 4.4).

**Table 1:** Characteristics of the subjects.

	Asymptomatic (*n* = 38)	Symptomatic (*n* = 20)
Age (years)	34.1 (12.4)	42.8 (15.4)
BMI (kg/m^2^)	24.4. (2.6)	24.7 (1.5)
Gender	Males (*n* = 19)	Males (*n* = 12)
Duration of symptoms (weeks)	–	3.1 (2.1)
Pain related disorder	–	*n* = 13
VAS (0–10/10)	–	4.6 (1.4)
Vertigo related disorder	–	*n* = 7
VVAS (0–10/10)	–	4.4 (2.4)

**Notes:**

Data represents mean and standard deviation unless otherwise noted.

VAS, visual analog scale; VVAS, visual vertigo analogue scale

### Concurrent validity

**Figure 4:**
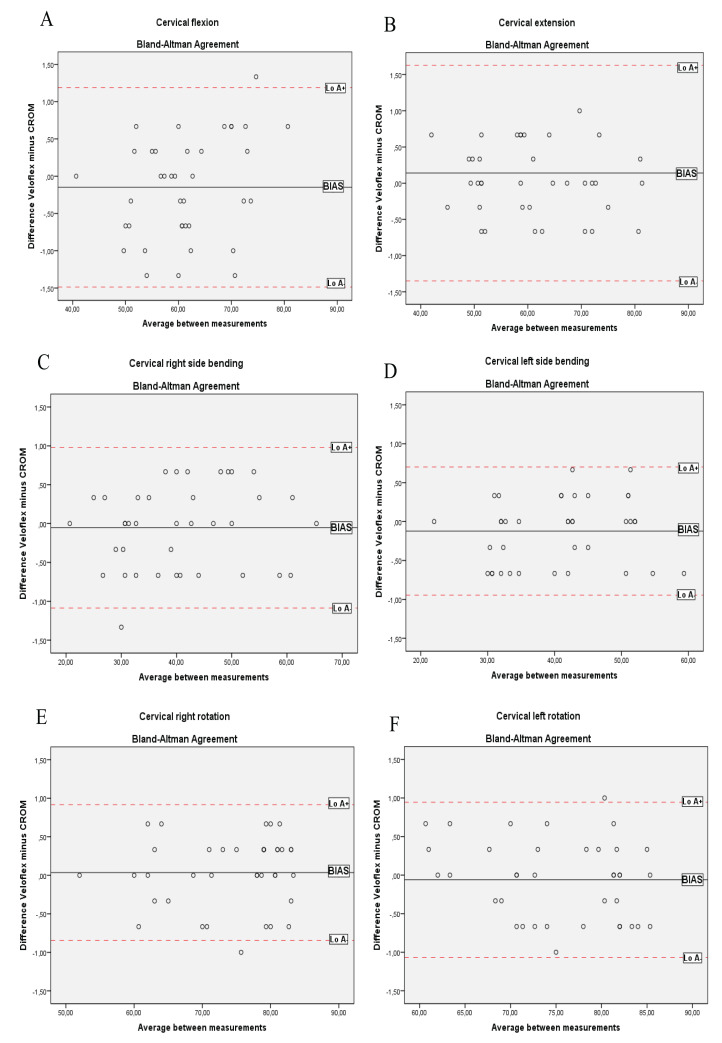
Bland–Altman plots for CROM and Veloflex during the cervical movements of: (A) flexion; (B) extension; (C) right side bending; (D) left side bending; (E) right rotation; (F) left rotation.

Cervical range of motion and VF showed excellent correlation for flexion/extension (*r*’s range = 0.993–0.998), side-bending (*r*’s range = 0.993–0.995) and rotation (*r*’s range = 0.960–0.974) movements (Table 2). ‘Bias’ (i.e. the differences between CROM and VF) for the six movements were close to 0°, and generally, these differences for each subject were located within the LoAs, as can be seen in the Bland–Altman plots (Fig. 4). Thus, both devices provided acceptable agreement of ROMs across movements with LoA− and LoA+ ≤2.30° and small mean ‘bias’ (≤1.29%) in all movements respect to measures with CROM.

**Table 2:** Validity between Veloflex and CROM to measure the cervical range of motion.

Cervical movement	Mean CROM (SD)	Mean veloflex (SD)	Pearson coefficient	LoA− (%)	LoA+ (%)	Mean difference (%)	SD difference (%)
Flexion	61.17° (8.60°)	61.35° (8.78°)	0.993	−1.83° (2.99%)	2.18° (3.56%)	0.17° (0.27%)	1.02° (1.66%)
Extension	61.22° (10.70°)	61.38° (10.7°)	0.998	−1.26° (2.05%)	1.60° (2.61%)	0.17° (0.27%)	0.73° (1.19%)
Right side bending	40.40° (11.01°)	40.56° (11.20°)	0.995	−2.02° (5%)	2.34° (5.79%)	0.16° (0.39%)	1.11° (2.74%)
Left side bending	40.63° (8.85°)	40.46° (8.90°)	0.993	−2.30° (5.66%)	1.96° (4.82%)	−0.17° (0.41%)	1.09° (2.68%)
Right rotation	74.30° (8.53°)	73.33° (7.51°)	0.974	−1.90° (2.55%)	2.15° (2.89%)	−0.96° (1.29%)	2.10° (2.82%)
Left rotation	75.49° (7.57°)	74.24° (7.24°)	0.960	−1.15° (1.52%)	1.43° (1.89%)	−1.24° (1.64%)	2.19° (2.90%)

**Note:**

SD, standard deviation; LoA, limit of agreement

### Reliability

Table 3 and 4 show intra- and inter-rater reliability analysis data for the VF in asymptomatic and symptomatic subjects. Intra- and inter-rater reliability was excellent (ICCs > 0.98) in both groups. SEMs ranging from 0.72% to 2.38% in all subjects and the MDC ranging from 1.47° to 2.60° in asymptomatic and from 1.22° to 2.18° in symptomatic subjects.

**Table 3:** Intra- and inter-rater reliability of Veloflex-measure the cervical range of motion in asymptomatic subjects.

Cervical movement	Intra-rater				Inter-rater			
	Retest (SD)	ICC (95% CI)	SEM (SEM%)	MDC	Test (SD)	ICC (95% CI)	SEM (SEM%)	MDC
Flexion	61.30° (8.70°)	0.99 [0.97–0.99]	0.86° (1.39%)	2.38°	61.35° (8.78°)	0.98 [0.97–0.99]	0.98° (1.59%)	2.71°
Extension	61.36° (10.71°)	0.99 [0.98–0.99]	0.74° (1.20%)	2.05°	61.37° (10.62°)	0.99 [0.98–0.99]	0.94° (1.53%)	2.60°
Right side bending	40.57° (11.19°)	0.99 [0.98–0.99]	0.78° (1.92%)	2.16°	40.56° (11.07°)	0.99 [0.98–0.99]	0.85° (2.09%)	2.36°
Left side bending	40.68° (8.90°)	0.99 [0.98–0.99]	0.79° (1.94%)	2.19°	40.46° (9.13°)	0.99 [0.98–0.99]	0.84° (2.07%)	2.33°
Right rotation	74.69° (7.16°)	0.99 [0.99–0.99]	0.67° (0.90%)	1.86°	74.24° (7.23°)	0.99 [0.98–0.99]	0.54° (0.72%)	1.50°
Left rotation	73.45° (7.69°)	0.99 [0.98–0.99]	0.74° (0.99%)	2.05°	73.33° (7.51°)	0.99 [0.99–0.99]	0.53° (0.72%)	1.47°

**Note:**

SD, standard deviation; ICC, intraclass correlation coefficient; CI, confidence interval; SEM, standard error of measurement; MDC, minimum detectable change

**Table 4:** Intra- and inter-rater reliability of Veloflex-measure the cervical range of motion in symptomatic subjects.

Cervical movement	Intra-rater				Inter-rater			
	Test (SD)/retest (SD)	ICC (95% CI)	SEM (SEM%)	MDC	Test (SD)	ICC (95% CI)	SEM (SEM%)	MDC
Flexion	49.32° (15.27°)/49.23° (14.99°)	0.99 [0.99–1]	0.47° (0.95%)	1.30°	49.29° (15.62°)	0.99 [0.99–0.99]	0. 60° (1.37%)	1.87°
Extension	49.50° (14.37°)/49.68° (14.03°)	0.99 [0.99–1]	0.44° (0.89%)	1.22°	49.62° (14.28°)	0.99 [0.99–0.99]	0.62° (1.25%)	1.73°
Right side bending	34.89° (10.46°)/35.40° (10.10°)	0.99 [0.99–0.99]	0.64° (1.82%)	1.77°	34.70° (10.56°)	0.99 [0.98–0.99]	0.79° (2.25%)	2.18°
Left side bending	32.61° (10.28°)/31.91° (9.43°)	0.99 [0.98–0.99]	0.75° (2.33%)	2.08°	32.61° (10.62°)	0.99 [0.98–0.99]	0.76° (2.38%)	2.12°
Right rotation	60.95° (19.83°)/60.81° (19.72°)	0.99 [0.99–1]	0.61° (1.01%)	1.71°	61.01° (19.70°)	0.99 [0.99–1]	0.61° (1.01%)	1.70°
Left rotation	61.27° (16.96°)/61.42° (16.94°)	0.99 [0.99–1]	0.53° (0.86%)	1.46°	60.89° (17.25°)	0.99 [0.99–0.99]	0.53° (0.87%)	1.48°

**Note:**

SD, standard deviation; ICC, intraclass correlation coefficient; CI, confidence interval; SEM, standard error of measurement; MDC, minimum detectable change

## Discussion

The present study showed that VF is a valid and reliable device to measure active CROMs. Moreover, it showed that basic training with VF for inexperienced examiners is enough to achieve reliable measures.

The differences between the correlation coefficients for all tests were minimal and also between the agreement’s statistics. Our correlations were higher, especially for the rotation, than previous studies that examined the validity of mobile applications using CROM as a reference measure (Tousignant-Laflamme et al., 2013). They argued that the CROM’s magnetic field could generate interference in the mobile and reduce its validity for measurements, which is an aspect not relevant for VF.

The intra-rater reliability coefficients were excellent and similar for all movements both in healthy participants and in those who had limited CROM. Other instruments such as the C-ROM (Audette et al., 2010), inclinometers (Kubas et al., 2017; Guidetti, Placentino & Baldari, 2017) mobile applications (Tousignant-Laflamme et al., 2013; Quek et al., 2014) or electronic devices (Law & Chiu, 2013; Song et al., 2018) also have high ICCs of intra-rater reliability for all cervical movements, except again for rotation movements with mobile applications which were poor. When the SEMs and MDC of these rotations were reported (Quek et al., 2014), they were around fifteen times higher than the VF’s SEM for rotation movements. In addition, absolute reliability of the VF was slightly better for all movements compared to the C-ROM (Audette et al., 2010), inclinometers (Kubas et al., 2017; Guidetti, Placentino & Baldari, 2017), mobile applications (Quek et al., 2014), electronic goniometer (Law & Chiu, 2013), or camera tracking systems (Song et al., 2018).

Inter-rater reliability also revealed high ICCs and low SEMs and MDCs. Once again, the differences between movements were minimal and absolute reliability of VF was slightly better compared to other devices (Law & Chiu, 2013; Kubas et al., 2017; Guidetti, Placentino & Baldari, 2017; Song et al., 2018). This study showed that the intra-rater reliability of the VF was similar to inter-rater for almost all movements. This finding is consistent with previous reliability studies of CROM measurement (Law & Chiu, 2013; Kubas et al., 2017; Guidetti, Placentino & Baldari, 2017; Song et al., 2018), in contrast with reliability of other body’s region measurements, where usually intratester reliability is slightly higher than intertester (Brosseau et al., 1997, 2001).

This study provides several research and clinical implications. First, CROM evaluation tools are important for the management of neck pain. These methods in turn must be reliable and easy to use for application in the clinical setting. This study has shown that VF is a tool with excellent reliability to measure CROM. In addition, with a basic training an inexperienced clinician can obtain reliable measurements. Second, not all ROM assessment tools can be used to give feedback of the joint path. Others, such as mobile applications that can be used for this purpose, require several accessories to place on the patient and hinder its clinical application. In contrast, our perception during VF’s is that it is an easy-to-use instrument to give feedback since it does not require complex accessories and has specific feedback software. Furthermore, the reliability of mobile applications to measure CROM is influenced by the smartphone model used (e.g. while iPhone 4 shows adequate intra-rater reliability, iPhone 3 shows poor inter-rater reliability) (Tousignant-Laflamme et al., 2013; Keogh et al., 2019), so its use to give feedback it could show the same problems from improper ROM measurements.

This study had several strengths. First, random errors were minimized by standardizing the procedures. To achieve this, the participants received the same instructions before each measurement and the same environment was maintained for all the data collection process. Second, the measurements were always taken with the two devices simultaneously or in the same order in the case of rotations. Thus, if the CROM increased with repetitions, this would not influence the results by maintaining a constant pattern of change among the participants. Third, we avoided an information bias, since the researcher who recorded the ROMs’ values was different from the examiner who made the measurements.

The main limitation is that there is the possibility that participants may have used substitution movements. The extension movement could be the most affected test when performing a thoracic extension to gain greater range. However, a standardized protocol, scripts, cueing and supervision were used to minimize compensations. One practice trial was also carried out of the tests to familiarize themselves with the procedures.

## Conclusions

The ROM assessment is essential in the clinical examination of neck pain. To this end, different tools are used, from more traditional such as the goniometer or inclinometers to more recent devices such as mobile applications or electronic systems. VF has been developed to track trajectories through a camera and evaluate the ROM cervical. Our study demonstrates that the VF is a valid and reliable measurement tool that can be easily used with a basic training.

## Supplemental Information

10.7717/peerj.11228/supp-1 Supplemental Information 1 Raw data.

10.7717/peerj.11228/supp-2 Supplemental Information 2 Codebook for the database.

## Additional Information and Declarations

### Data Availability

The following information was supplied regarding data availability: The raw measurements and the codebook are available in the Supplemental Files.
