# Supplementary material for: Validity and reliability of Veloflex to measure active cervical range of motion in asymptomatic and symptomatic subjects
Source: PeerJ. 2021 Apr 5;9:e11228. doi: 10.7717/peerj.11228 (PMC8029663; doi:10.7717/peerj.11228)
Supplement: Supplemental Information 2 [file peerj-09-11228-s002.docx]

Variable Group:

0- Symptomatic

1- Asymptomatic

Variable gender:

1- Male

2-Female
